# Exuberant long noncoding RNA expression may sculpt Igh locus topology

**DOI:** 10.3389/fimmu.2025.1678105

**Published:** 2025-11-24

**Authors:** Ellen B. Drake, Sarah Naiyer, Xinyan Qu, Khalid Bhat, Hammad Farooq, Mark Maienschein-Cline, Jie Liang, Amy L. Kenter

**Affiliations:** 1 Department of Microbiology and Immunology, University of Illinois College of Medicine, Chicago, IL, United States; 2 Center for Bioinformatics and Quantitative Biology, and Department of Biomedical Engineering, University of Illinois Colleges of Engineering and Medicine, Chicago, IL, United States; 3 Research Informatics Core, Research Resources Center, University of Illinois, Chicago, IL, United States

**Keywords:** progenitor B cells, V(D)J recombination, IgH locus, chromatin folding, long non-coding RNA

## Abstract

Diverse Igh repertoires require successful V(D)J recombination allowing B cell receptor expression and Ig secretion for humoral immune responses. Igh locus contraction has been implicated in generating spatial proximity between distal V_H_ segments and the recombination center via cohesin mediated loop extrusion. However, it remains unclear why some distal V_H_ segments recombine with high frequency while other more proximal V_H_ are rarely used. Long non-coding RNAs (lncRNAs) have emerged as regulators of cellular development, differentiation and gene expression. Here we report exceptionally high expression of lncRNAs at the Igh locus and other AgR loci engaged in V(D)J recombination. A tight correlation was found between positions of multi-exonic lncRNAs, Igh enhancers and chromatin loop anchors. We propose an integrated model of factors including lncRNAs and loop extrusion in determining Igh locus topology and V_H_ gene usage during recombination.

## 1 Introduction

Adaptive immune responses use antigen receptors (AgRs) expressed on B and T lymphocytes to protect against a multitude of pathogens. Each mature B cell expresses a unique immunoglobulin (Ig) receptor containing two identical Ig heavy (H) chains and two identical light (L) chains (Igκ or Igλ). The variable regions of IgH and IgL chains are assembled from V, D, J and V, J segments, respectively, by V(D)J recombination in B cell progenitors (1, 2). This process is mediated by the lymphocyte-specific RAG1/2 recombinase that creates DNA breaks at recombination signal sequences (RSSs) flanking each V, D and J segment (1, 2). V(D)J recombination for the Igh locus involves a two-step process during which one of 8–12 D_H_ and one of 4 J_H_ gene segments are rearranged to generate a DJ element that is then recombined with one of ~100 functional V_H_ genes in each pro-B cell (3). V_H_ genes rearrange at very different intrinsic frequencies to produce a quasi-random V_H_ gene usage profile (4–6). Evaluation of V germline transcript levels, chromatin accessibility, transcription factor (TF) binding, RSS quality, and epigenetic profiles indicates that no single variable fully accounts for skewed V_H_ gene usage (4–6). The observation that V_H_ gene families are clustered in the locus (3) and display characteristic features regarding the position of bound CTCF (7, 8), regionally distributed histone modifications, RNAPII and TF binding (4, 5) suggests that V_H_ segment selection for each group may be differentially regulated. Nevertheless, the mechanisms underlying unequal V_H_ gene rearrangement frequencies remain unresolved.

Mammalian chromosomes at the Mb scale are organized into topological associating domains (TADs) that span spatial neighborhoods of high frequency self-associating chromatin contacts (9–11). The Igh locus is contained within a 2.9-Mb TAD that is divided into three subTADs and V_H_ gene families are regionally arrayed within this spatial genomic context (12, 13). TADs are frequently anchored by motifs bound by the CTCF architectural protein and its interaction partner, cohesin (14, 15). Cohesin maintains TAD structure by progressively extruding DNA loops until encountering a dominant obstacle such as a bound convergently oriented CTCF which halts movement and anchors DNA loops in mammalian cells (16, 17). The loop extrusion model explains how enhancers (Es) can processively track arrays of promoters (Prs) through the extruding chromatin loop over long genomic intervals (16–19).

The Igh locus is in an extended configuration in non-B cells and lymphoid progenitors and becomes contracted on both alleles in pro-B cells when V->DJ rearrangements occur (20, 21). Igh locus contraction is cohesin dependent as acute depletion of WAPL (Wings apart like protein), the cohesin release factor (22, 23), leads to both increased chromatin loop formation and locus contraction. Relatedly, TF PAX5 mediates locus contraction and distal V_H_ gene recombination (20, 24) through its suppression of Wapl transcription (22) linking these processes. The Alt group has established the RAG scanning model to explain how distal V_H_ segments in contracted Igh loci become spatially situated proximal to the V(D)J recombination center through a linear tracking mechanism with parallels to loop extrusion (reviewed in (25, 26)).

V_H_ gene choice may depend on the dynamics of cohesin mediated loop extrusion which leads to variable loop lengths and different final end-points (27, 28). Impediments to loop extrusion are likely to influence the local-regional propensity for chromatin loop formation and V_H_ segment engagement in recombination (25). Antisense RNA expression has been detected at discrete sites within the Igh locus (29), however, the extent to which this expression modulates Igh locus function is unknown. In this context, it is intriguing to consider the potential regulatory influence of long non-coding (lnc) RNAs that can originate from thousands of loci genomewide (reviewed in (30–32)). LncRNA functions include modulation of chromatin architecture, transcription, RNA processing and splicing (33–35), within the B cell lineage (36), and in processes such as V(D)J recombination and class switch recombination (CSR) (37, 38). Here we report highly elevated expression of lncRNAs in B- and T-cell progenitors undergoing V(D)J recombination. We document hundreds of Igh associated lncRNAs that cluster to TAD anchors and enhancers of pro-B cells. We provide a perspective on the interplay of Igh lncRNAs, locus structure and V_H_ gene choice during V(D)J recombination.

## 2 Materials and methods

### 2.1 Mice, pro-B cells and cell lines

Rag2^-/-^ mice on the C57BL/6 background were purchased from Jackson Laboratories or maintained in colonies at the University of Illinois College of Medicine. All procedures involving mice were approved by the Institutional Animal Care Committee of the University of Illinois College of Medicine in accordance with protocols approved by the UIC Institutional Animal Care and Use Committees. Mice were housed in sterile static microisolator cages with water bottles and on autoclaved corncob bedding. Irradiated food was (Envigo 7912), and autoclaved water were provided *ad libitum*. Mice receive autoclaved nesting material to enrich their environments. Cage bedding is changed in either a biosafety cabinet or a HEPA filtered animal transfer station at least weekly. Housing density and cage size are consistent with the recommendations of the *Guide for the Care and Use of Laboratory Animals*. Mouse rooms received the standard photoperiod of 14 hours of light and 10 hours of darkness. The ambient temperature and humidity of the rodent housing rooms are consistent with the recommendations of the *Guide for the Care and Use of Laboratory Animals*. Rag2^-/-^ pro-B cells were isolated from BM using anti-CD19 coupled magnetic beads (Miltenyi, catalogue number 130-121-301, RRID: AB_2827612) and cultured in the presence of IL7 (1% vol/vol supernatant of a J558L cell line stably expressing IL7) for 4 days. The Abelson-MuLV transformed (Abl-t) pro-B cell line, 445.3 (Rag1^-/-^) on the C57Bl/6 background was kindly provided by Dr. B. Sleckman (University of Alabama at Birmingham) (39). The 445.3.11 subclone from the Abl-t 445.3 line was cultured in RPMI 1640 (Corning, 15040CV), 10% (v/v) FBS, 4mM glutamine (Gibco), 1mM sodium pyruvate (Gibco), 1X nonessential amino acid (Gibco), 5000 units/ml Penicillin and 5000 mg/ml Streptomycin (Gibco), 50 mM b-mercaptoethanol (Sigma) and maintained at approximately 5x10e5 cells/ml. Splenic T cells were isolated using Mouse T Cell Enrichment Columns (MTCC-5; R&D Systems) and cultured (5X10e5 to 1X10e6 cells/ml) in RPMI 1640 and glutamine (4 mM) with Penicillin- Streptomycin supplemented with FCS (10% v/v), and activated with Con A (5 ng/ml; 15324505; MP Biomedicals).

### 2.2 Genome-wide LncRNA annotation, ChIP-seq data sets and RT-PCR assays

#### 2.2.1 LncRNA annotation

Published RNA-seq datasets from Rag1^-/-^ pro-B cells (GEO accession numbers: GSM1897405, GSM1897406, GSM1897407) (40), pre-B cells from Rag1^-/-^μ^+^ mice bearing a rearranged Igμ transgene (GEO accession numbers: GSM1897411, GSM1897412, GSM1897412) (41), RAG^-/-^YY1^f/f^ x Mb1-Cre pro-B cells (GEO accession numbers: GSM1897408, GSM1897409, GSM1897410) (41), Rag1^-/-^ thymocytes bearing a TCRβ transgene (GEO accession numbers: GSM1701762, GSM1701763, GSM1701764) (42), Rag1^-/-^ CD3 activated DP thymocytes (GEO accession numbers: GSM1701765, GSM1701766, GSM1701767) (42), Rag2^-/-^ CD3 activated DP thymocytes (GEO accession numbers: GSM1701768, GSM1701769, GSM1701770) (42) were analyzed. Reads were concatenated from two-three independent samples and mapped reads were aligned to the genome (mm10) using STAR (version 2.5.2b, default settings). Transcript assembly was performed with StringTie (version 2.0, default settings) and transcripts (≥300 nucleotides, FPKM ≥0.3) were documented (**Supplementary Data Sheets 1–6**). LncRNAs derived from the Igh locus in Rag1^-/-^ pro-B cells were annotated (**Supplementary Data Sheet 7**). The mouse genome (mm10) was subdivided into 100kb bins. Contiguous bins expressing lncRNA transcripts were merged into windows. Window boundaries were defined as occurring when >2 bins lacking lncRNA transcripts. Each window was analyzed for the number of aligned lncRNA transcripts (hits), transcript length, weighted coverage (total transcript length x expression (FKPM)) and an overall window score was calculated by taking the sum of the three criteria scores for each window (**Supplementary Data Sheets 8–13**). LncRNA transcript genomic coordinates were converted to mm9 for visualization. **Supplementary Data Sheets 1–13** are available at: https://uofi.box.com/s/1lynmquf08ct0qtp4swxg713foaa34v5.

#### 2.2.2 ChIP-seq data sets used to analyze the Igh locus in pro-B cells

Public ChIP-seq data sets were analyzed for Rag deficient pro-B cells: H3K27ac (GEO accession number: GSM2255552) (43); H3K4me1 (GEO accession number: GSM546527) (44); p300 (GEO accession number: GSM987808) (44); CTCF (GEO accession number: GSM1156665) (4); Rad21 (GEO accession number: GSM1156667) (4); E2A (GEO accession number: GSM546523 (44); RNA Pol II (GEO accession number: GSM1156660) (4).

#### 2.2.3 RT-PCR assays

RNA extraction was performed from BM Rag2^-/-^ pro-B cells (2-3x106 cells) using TRIzol (Life Technologies) and then treated with DNase with the DNase I kit (Invitrogen) all according to manufacturer’s instructions. CDNA synthesis was performed using RNA (1 µg) and the SuperScript II Reverse Transcriptase kit (Invitrogen) per the manufacturer’s instructions. RT-PCR assays for lncRNA transcripts were carried out using 10x Platinum Taq reaction buffer (2.5.μl), 50mM MgCl2 (0.75μl), 10mM dNTPs (0.5μl), forward (0.5μl) and reverse (0.5μl) primer (10mM), cDNA (2μl), and 0.1μl Platinum Taq DNA polymerase (Invitrogen) (5U/μl) in a 25μl reaction volume. A touchdown PCR was performed: 95C, 3’; 10 cycles (95C, 30” 64C, 45”; -1 degree C each cycle; 72C, 30”), 25 cycles (95C, 30”; 54C, 45”; 72C, 30”; 72C, 5’) 4C hold. PCR products were visualized on a 1.5-2% agarose gel electrophoresis. Primers are listed in **Supplementary Table 1**.

### 2.3 Hi-C library construction and analyses

Genome-wide *in situ* HiC libraries were constructed from Rag2^-/-^ pro-B cells expanded in IL7 for 4–5 days using Arima Hi-C kits (Arima Genomics, San Diego, CA) as recommended by the manufacturer, as previously described (45). *In situ* Hi-C was performed using two biological replicates that yielded a minimum of 1.3 billion read pairs and 0.72 billion from pro-B cells of each genotype (GEO Accession No. GSE201357) (**Supplementary Table 2**). Published *in situ* Hi-C data sets for mouse embryonic fibroblasts (MEFs) were constructed with Arima Hi-C kits (GEO Accession No. GSE113339) and data was handled in parallel with the pro-B cell data. *In situ* Hi-C data was processed using the Juicer pipeline (v.1.5), CPU version (46). Extraction of virtual 4C interaction matrices: Hic files derived using Juicer tools were used to generate virtual 4C viewpoints from dumped matrices generated in Juicebox. KR normalized observed read matrices were extracted at 10kb resolution. The biological replicates had stratum adjusted correlation coefficient (SCC) (47) greater than 0.9 and were merged. The interaction profile of virtual 4C were plotted by running a rolling window of 30kb with a 10kb slide. Generation of Hi-C difference maps: Experimentally measured Hi-C contact matrices of individual replicate and merged samples were quantile normalized against the Hi-C contact matrices of the uniformly sampled random ensemble of the corresponding cell type as previously described (45).

### 2.4 3C library construction and analysis.

3C chromatin was prepared from CD19^+^ IL7 expanded Rag2^-/-^ pro-B cells, the Abl-t 445.3.11 line and ConA activated splenic T cells as previously described (45, 48). 3C library construction using Hind III and assays for the Igh locus were performed as described earlier (49, 50). Quantitative PCR (qPCR) in combination with 5’FAM and 3’BHQ1 modified probes (IDT) was used to detect of 3C products and primers were designed using Primer Express software (ABI) (**Supplementary Table 3**). Primer and probe optimization were carried out according to the manufacturer’s recommendations, (http://www3.appliedbiosystems.com/cms/groups/mcb_support/documents/generaldocuments/cms_042996.pdf). P values were calculated by using two-tailed Student’s t test. In all cases p values are shown.

### 2.5 ATAC-seq and analysis

ATAC-seq libraries were constructed from purified CD19+ Rag2^-/-^ pro-B cells (5x10^4^/sample) that had been expanded in IL7 for five days using the Nextera DNA Library Prep Kit (Illumina) according to the manufacturer’s instructions with the following exception. Cells were lysed in cold C1 buffer (50ml) (10 mM Tris [pH 7.5], 5 mM MgCl2, 11% sucrose, 1% Triton X-100) and incubated for 10 min on ice to generate nuclei. DNA was purified using the Zymo DNA Clean & Concentration Kit (D4013). DNA tagmentation fragments were amplified as specified (51) and used for Nextera library construction followed by NGS analysis. Adaptor sequences were trimmed using *SeqPurge* (v2019_11) and trimmed reads were mapped to mm10 mouse genome assembly using *Bowtie2* (v2.2.9) with settings *– very-sensitive -X 2000*. PCR duplicates were removed using *Picard* (v2.21.8) *MarkDuplicates REMOVE_DUPLICATES=true VALIDATION_STRINGENCY=LENIENT*. Reads with MAPQ scores below 30 were purged using *samtools* (v1.9) *view* with settings *-b -q 30 -f 2 -F 1804*. The samples 1) Rag2KO_1 had 34923628 total paired-end reads of which 98.4% were mapped and 2) Rag2KO_2 had 68921951 total paired-end reads of which 97.2% were mapped. Peak calling and sample normalization were carried out as described (52). To facilitate comparison of peaks across samples, the MACS2 peak scores (-log10(p-value)) for each sample were converted to a score per million (SPM) by dividing each peak score by the sum of all of the peak scores in a sample divided by 1 million. Sample peak sets were merged, less significant overlapping peaks removed, and remaining peaks were filtered for those that were observed in at least two samples with an SPM value 2. To generate peak-by-sample count matrices, ATAC fragment counts within each peak were normalized by the number of inserts intersecting nucleosome-depleted promoter regions (-300 bp to +100 bp relative to transcriptional start-sites). ATAC-seq library data are available (GEO accession number: GSE214081). ATAC-seq files were converted to genome coordinates mm9 for visualization.

## 3.0 Results

### 3.1 Novel lncRNAs are highly enriched at the Igh locus in pro-B cells

We analyzed RNA-seq datasets to identify novel lncRNA transcripts expressed in Rag1^-/-^ pro B cells (**Supplementary Data Sheet 1**) (40). The mouse genome was subdivided into 100kb bins which were assessed for the number of unannotated aligned transcripts (hits, >300nt), transcript length, weighted coverage (total transcript length x expression (FKPM)). An overall bin score was calculated by taking the sum of the three criteria scores. Contiguous bins containing expressed lncRNA transcripts were merged into windows and window boundaries were defined as >2 consecutive 100kb bins lacking these transcripts. Windows were ranked for lncRNA expression genomewide using the three criteria scores. The Igh locus was encompassed in a single window and attained the top ranked overall score demonstrating that this region is exceptionally enriched with expressed lncRNAs in Rag1^-/-^ pro-B cells (**Table 1**). In contrast, the Igλ and Igκ loci, which do not undergo rearrangement until the pre-B cell stage of development, attained window scores of 224 and 269, respectively suggesting a strong correlation between locus specific lncRNA expression and V(D)J recombination potential (**Supplementary Data Sheet 2**) (**Table 1**).

**Table 1:** The Igh locus is enriched for lncRNA in pro-B cells.

Sample	Genomic coordinates (mm10)	Overall window rank^#^	Locus^$^
Rag1^-/-^ proB 1816 total windows	chr12:112400000-116700000	1**	Igh
	chr13:18600000-25000000	70**	Tcrg
	chr14:54000000-57000000	132*	Trad;Trad_pt4*
	chr14:51800000-53200000	220	Trad;Trad_pt1;Trad_pt2
	chr16:18800000-20700000	224	Igl
	chr6:70700000-74300000	269	Igk
	chr6:40000000-41700000	478	Trb
	chr6:67000000-69200000	537	Igk
	chr6:69700000-70100000	1149	Igk
Rag1^-/-^YY1^-/-^ pro-B 1535 total windows	chr12:112600000-116500000	6**	Igh
	chr14:54200000-56100000	207	Trad;Trad_pt4^
	chr16:16800000-19300000	249	Igl
	chr6:41600000-41700000	311	Trb
	chr6:41100000-41200000	324	Trb
	chr6:40300000-40800000	464	Trb
	chr13:19200000-19800000	494	Tcrg
	chr6:67800000-68400000	772	Igk
	chr6:70700000-70900000	931	Igk
	chr6:68800000-68900000	1474	Igk
	chr6:69300000-69400000	1510	Igk
	chr6:70200000-70300000	1516	Igk
Pre-B (Rag1^-/-^ Ig*μ* Tg) 1576 total windows	chr12:114800000-115800000	18**	Igh
	chr12:112600000-113600000	22**	Igh
	chr14:54200000-55900000	38**	Trad;Trad_pt4^
	chr16:16800000-19400000	99*	Igl
	chr6:67200000-68900000	304	Igk
	chr6:69600000-72500000	306	Igk
	chr14:51800000-52400000	426	Trad;Trad_pt1
	chr14:53500000-53600000	1097	Trad;Trad_pt3
	chr6:41600000-41700000	1169	Trb
	chr13:19300000-19500000	1219	Tcrg
	chr6:40300000-40800000	1309	Trb
	chr12:114400000-114500000	1328	Igh
	chr6:41100000-41200000	1371	Trb

# Overall window score was assigned by taking the sum of the criteria scores (number of aligned lncRNA transcripts (hits, >300nt), transcript length, weighted coverage (total transcript length x expression (FKPM)). Windows ranked in the top 5% and 10% are denoted with ** and * respectively.

$ Genomic windows containing an antigen receptor (AgR) locus are shown. Windows that contain the 5’ most edge of the TCR*α*δ (Trad) locus but the lncRNAs are coming from outside of this locus are denoted (^).

To test the specificity of lncRNA transcript expression in the Igh locus of pro-B cells we evaluated three independent RNA-seq datasets from double positive (DP) CD4^+^CD8^+^ thymocytes that are incapable of Igh rearrangements. LncRNA expression at the Igh locus was greatly diminished in DP thymocytes compared to that found in pro-B cells demonstrating lineage specificity (**Supplementary Data Sheets 3–5**) (**Table 2**). Furthermore, the Igh locus was split into several windows indicating a loss of window contiguity and underscoring the greatly diminished lncRNA expression at this locus in DP thymocytes (**Tables 1**, **2**). These findings highlight a correlation between Igh locus activation and lncRNA expression in lymphocytes.

**Table 2:** TCR loci are enriched for lncRNAs in DP T cells.

Sample	Genomic coordinates (mm10)	Window rank^#^	Locus^$^
Rag2^-/-^ CD3-stimulated thymocytes 1525 windows	chr14:53600000-55200000	2**	Trad;Trad_pt3;Trad_pt4
	chr6:41100000-41700000	133*	Trb
	chr13:18800000-19400000	619	Tcrg
	chr12:116000000-116500000	768	Igh
	chr6:70700000-70800000	1364	Igk
Rag1^-/-^ CD3-stimulated thymocytes 1513 windows	chr14:53000000-55200000	19**	Trad;Trad_pt2;Trad_pt3;Trad_pt4
	chr6:41100000-41700000	136*	Trb
	chr12:112600000-113500000	221	Igh
	chr12:116000000-116500000	314	Igh
	chr13:18800000-19400000	710	Tcrg
	chr6:70700000-70800000	1329	Igk
	chr12:114500000-114600000	1495	Igh
Rag1^-/-^ TCRβ Tg 1509 windows	chr14:53000000-55200000	34**	Trad;Trad_pt2;Trad_pt3;Trad_pt4
	chr6:41100000-41700000	59**	Trb
	chr12:112600000-113500000	86*	Igh
	chr13:18800000-19400000	844	Tcrg

# Overall window score was calculated by taking the sum of the criteria scores (number of aligned lncRNA transcripts (hits, >300nt), transcript length, weighted coverage (total transcript length x expression (FKPM)). Windows ranked in the top 5% and 10% are denoted with ** and * respectively.

$ Genomic windows containing antigen receptor (AgR) loci are shown.

#### 3.1.1 LncRNA expression at AgR loci is linked to V(D)J potential

To explore the proposition that elevated lncRNA expression may be a general feature of AgR loci engaged in V(D)J recombination we examined TCR loci in DP thymocytes and in B cell progenitors for lncRNA transcripts. RAG1/2 recombinase is first expressed in double negative (DN) CD4^-^CD8^-^ thymocytes whereupon the *Tcrg*, *Tcrd* and *Tcrb* all undergo recombination (53, 54). *Tcrb* recombination facilitates assembly of the pre-TCR containing a rearranged β gene, commitment to the αβ T-cell lineage and differentiation into the DP stage of development (55). The *Tcra* locus is capable of V(D)J rearrangement in DP TCRβ Tg+ thymocytes (56). Using three independent RNA-seq data sets we examined lncRNA expression in DP CD3 stimulated thymocytes and in thymocytes expressing the TCRβ Tg. The large *Tcrad* locus achieved overall window scores of 2, 19 and 34 for lncRNA expression and was encompassed in single large windows (**Table 2**). In contrast, the Tcrad locus was ranked 220 overall and was split into several windows in pro-B cells where it does not recombine (**Table 1**). These observations suggest that elevated expression of lncRNA transcripts is a feature of AgR loci capable of engaging in V(D)J recombination. We conclude that expression of novel lncRNAs is elevated in AgR loci and correlated with V(D)J recombination potential in a lineage specific fashion.

#### 3.1.2 Igh locus contraction is not required for elevated lncRNA expression

Igh locus contraction facilitates distal V_H_ gene usage during V->DJ recombination and is dependent on expression of the B lineage determining TFs, PAX5 and YY1 (20, 57). In the absence of locus contraction only V_H_ genes in close proximity to the recombination center located in the Eμ-D-J cluster engage in V->DJ recombination whereas V_H_ segments at more distal positions remain unrearranged (58). Locus contraction may also influence gene expression by providing V_H_ and lncRNA exons access to distal Igh enhancers spanning the locus (45). To test the proposition that elevated lncRNA expression requires locus contraction we evaluated RNA-seq data sets from Rag1^-/-^YY1^-/-^ pro-B cells (**Supplementary Data Sheet 6**) (41). In this case, the Igh locus coalesced into a single window and achieved an overall window score of 6 suggesting that failure to undergo robust locus contraction does not significantly impair locus affiliated lncRNA expression and implies that lncRNA expression does not require proximity to Eμ (**Table 1**).

### 3.2 Igh associated lncRNAs are both mono- and multi-exonic

Numerous Igh associated noncoding (nc) RNAs are detected in Rag1^-/-^ pro-B cells when no minimum length requirement is applied (**Supplementary Data Sheet 7**). Characterization of Igh associated lncRNAs (>300 nt) indicates that most are mono-exonic (218/242) and are arrayed in both the sense (120/218) and antisense (98/218) orientations with little overlap (**Supplementary Data Sheet 7**). In contrast, most multi-exonic lncRNAs (24/242) are in the antisense orientation (20/24) (**Supplementary Data Sheet 7**). Ample evidence indicates that some lncRNAs direct chromatin remodeling complexes to specific genes and regulate pluripotency, neurogenesis and brain development (59–64). Therefore, we asked whether lncRNAs overlap with- or are in proximity (within 500 bp) to-V_H_ genes and thereby influence V_H_ usage during V(D)J recombination. We first considered V_H_-lncRNA overlap, as this configuration is most likely to influence chromatin accessibility. We find that 144/180 V_H_ are used in V(D)J recombination (45). However, only 40/180 V_H_ genes overlap with lncRNAs and of these 32/144 are used in V(D)J recombination indicating no overall correlation between V_H_-lncRNA overlap with V_H_ gene usage. Nevertheless, those minority V_H_ genes overlapped with lncRNAs are predisposed for usage in recombination. Additionally, no correlation was found between lncRNA proximity within 500 bp of V_H_ genes leading to V_H_ usage in V->DJ recombination leaving open the question of lncRNA function in the Igh locus.

### 3.3 Multi-exonic lncRNAs cluster to Igh architectural anchors and enhancers

**Figure 1:**
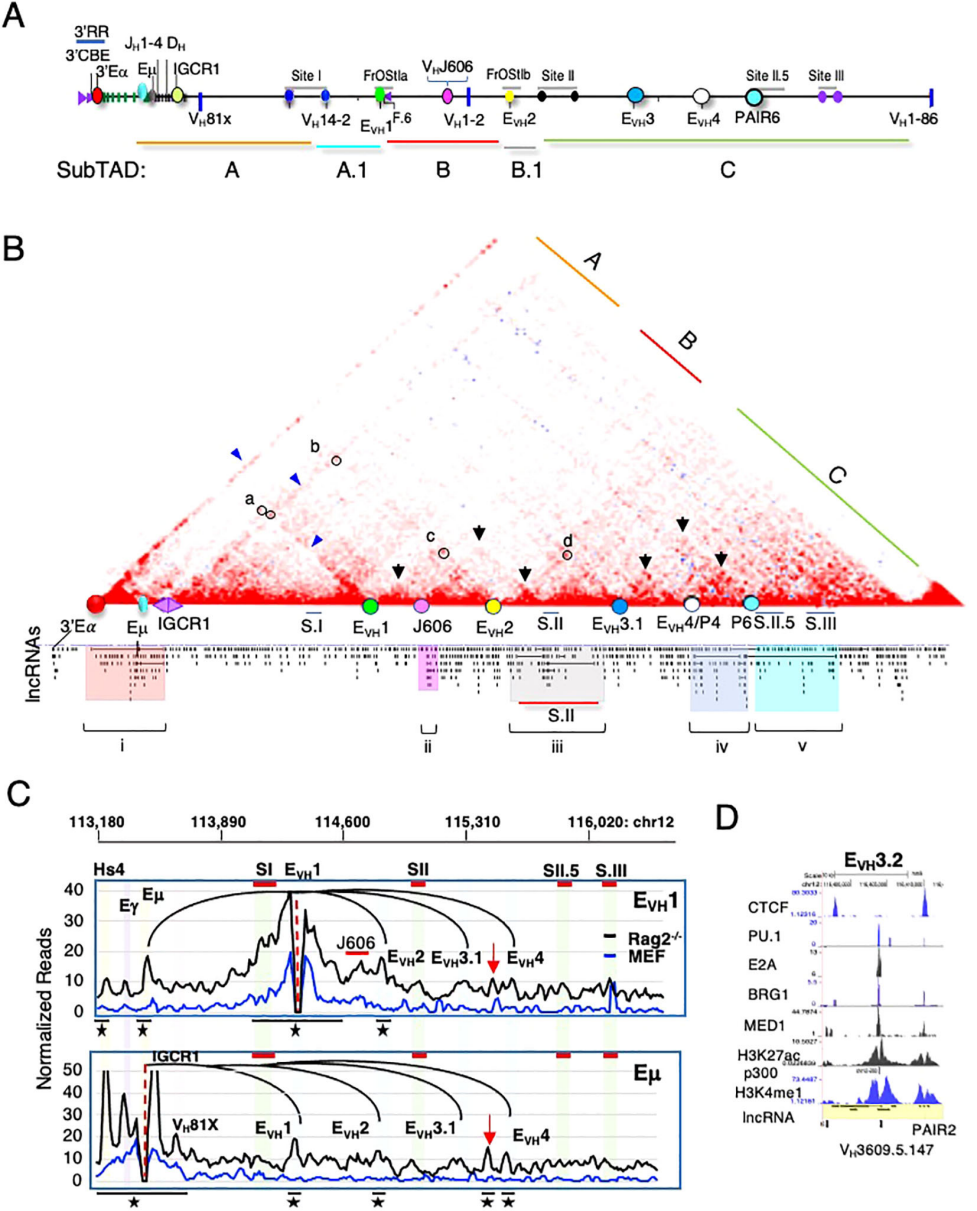
Igh multi-exonic lncRNAs focus to architectural loop anchors. Genomic coordinates, mm10. **(A)** Diagram of the Igh locus. Regulatory elements shown are annotated on the HiC heatmap below. **(B)** Hi-C difference heatmap (chr12:113220000-116010000)(10kb resolution) with regulatory elements (colored dots) from Rag2^-/-^ pro-B cells (n=2). DNA elements (3’Eα, red dot; CBEs, purple arrows; Eγ, teal dot; Eµ, gray oval; E_VH_1 (chr12:114182400-114183200), E_VH_2 (chr12:114609100-114609900), E_VH_3 (chr12:115023400-115024300), E_VH_4 (chr12:115257500-115258400); C_H_ region genes (green bars); D_H_ and J_H_ exons (black bars); selected V_H_ genes. Annotated lncRNAs are arrayed below the Hi-C heatmap and clusters i-v highlighted. **(C)** Igh enhancer-promoter hub. Virtual 4C interactions (mm10) were extracted from KR normalized Hi-C data sets from Rag2^-/-^ pro-B cells (black lines) and murine embryonic fibroblasts (MEF) (blue lines). *Top*: Genomic coordinates with E_VH_1 and Sites (S) I, II, II.5 and III. E_VH_1 4C anchor (dashed vertical line) in a 30 kb running window analysis with 10 kb steps from merged biological replicates with E_VH_1 interactions (black arcs). Stars (top 15%) of locus-wide interactions. Putative distal enhancer (red arrow). Upper panel: E_VH_1 viewpoint. Lower panel: Eμ viewpoint. **(D)** Putative distal enhancer, termed E_VH_3.2 characterized for histone modifications, Pol II co-activators and TF binding in ChIP-seq assays and lncRNA expression derived from RNA-seq analyses.

NcRNAs often colocalize with structural elements within TADs (reviewed in (30, 65). We examined Hi-C datasets from Rag2^-/-^ pro-B cells to explore the relationship of Igh locus conformation with lncRNAs. Rag deficiency ensures that the Igh locus remains in a germline configuration and is comparable to other cell types. Hi-C difference maps were constructed by subtracting mouse embryonic fibroblast (MEF) from Rag2^-/-^ contacts to identify pro-B specific interactions (12, 45). The Hi-C difference map for Rag2^-/-^ pro-B cells shows a subTAD structure that is essentially identical to that found in Rag1^-/-^ pro-B cells indicating a high degree of reproducibility (**Figure 1B**) (45). The Igh TAD is structurally divided into three subTADs and two additional mini-subTADs A.1 and B.1 that are anchored at Eμ, IGCR1, Sites I, II, II.5, III, Friend of Site Ia (FrOStIa) and FrOStIb and distal V_H_ enhancers (**Supplementary Figures 1A, B**) (12, 45). Seven Igh enhancers include three at the 3’ end (3’Eα, Eγ and Eμ) and four V_H_ distal enhancers (E_VH_1-4) (45). E_VH_1 and E_VH_2 are located within FrOStIa and FrOStIb, respectively (**Figure 1A**) (**Supplementary Figure 1**). E_VH_3.1 marks the boundary between the clustered and interspersed V_H_J558 family genes and E_VH_4 is adjacent to Pax5-activated intergenic repeats (PAIR) 4 (**Figure 1A**). PAIR comprise a series of fourteen elements located in subTAD C that bind TF PAX5, E2A, and CTCF in pro-B cells (66, 67). PAIR6 and PAIR 11 co-locate with Sites II.5 and III, respectively (**Figure 1A**)(**Supplementary Figure 1**).

The Hi-C difference heatmaps from Rag2^-/-^ pro-B cells reveal asymmetric architectural stripes (blue arrowheads) and dots (black circles) representing loop domains that combine to define the conformational state of the Igh TAD (**Figure 1B**) (45). Stripes originating from 3’Eα, IGCR1 and from E_VH_1 are particularly evident (**Figure 1B**). Topological stripes are generated by loop extrusion when one subunit of dimeric cohesin stalls while the second subunit proceeds along chromatin in an ATP dependent fashion to form multiple contacts (14, 16, 17, 68). In addition to distinct chromatin loops visualized as dots (black circles), loops can also be folded into domains containing a plethora of intra-loop contacts (black arrows) such as found between Site I (S.I)-E_VH_1, E_VH_1-V_H_J606 (a V_H_ subfamily), and V_H_J606-E_VH_2, E_VH_2-S.II, S.II-E_VH_3.1, E_VH_3.1-E_VH_4/PAIR4, and E_VH_4/PAIR4-PAIR6/SII.5 and that structure the locus (**Figure 1B**). Igh associated lncRNAs are arrayed below the Hi-C map to highlight the disposition of architectural elements with lncRNA expression in cis (**Figure 1B**). Five prominent clusters of mono-exonic and spliced lncRNAs are evident including those that span from Cγ2b and Eγ through Eμ and the D_H_-J_H_ segments (cluster i), V_H_J606 gene family (cluster ii), S.II (cluster iii), PAIR4-PAIR6 (S.II.5) (cluster iv) and PAIR11 (cluster v) and highlight a remarkable correlation between major Igh architectural elements and nested lncRNA positions (**Figure 1B**).

#### 3.3.1 V_H_ distal enhancers and PAIR elements participate in an enhancer interactome

Our earlier studies in the Rag1^-/-^ genetic background demonstrated the presence of an Igh enhancer hub (45) that is thought to help balance dynamic transcription (69). Deletion of E_VH_1 was found to alter the composition of the enhancer interactome as well as to reduce V_H_ gene usage in a defined chromatin domain (45). Here virtual 4C analyses were used to visualize Eμ and E_VH_1 centered chromatin contacts over long genomic distances and to further assess the relationship of these enhancers with lncRNA expression in Rag2^-/-^ pro-B cells. High frequency interactions defined as the top 15 (star) percent of all contacts anchored at E_VH_1 and Eμ in both biological replicates for Rag2^-/-^ (black symbols) pro-B cells were identified (**Figure 1C**). Virtual 4C contact maps indicate that the E_VH_1 viewpoint interacts with Eμ and the distal enhancers, E_VH_2, E_VH_3.1, E_VH_4 in Rag2^-/-^ pro-B cells (black trace) and are absent in MEF (blue trace) and a similar contact profile was detected for the Eμ viewpoint indicating pro-B cell specificity (**Figure 1C**). Both E_VH_1 and Eμ interact with a new element (red arrow) which we term E_VH_3.2 (see below) in Rag2^-/-^ pro-B cells and not in MEF (**Figures 1C, D**). Earlier DNA fluorescence *in situ* hybridization (FISH) studies detected Eμ-E_VH_1-E_VH_2 interactions directly demonstrating the presence of a multi-way enhancer hub (45). The Igh enhancer interactome may serve to stabilize locus contraction by virtue of E-E-E interactions generated through cohesin mediated loop extrusion.

#### 3.3.2 Spliced lncRNAs are expressed from Igh enhancers and a subset of V_H_ promoters

**Figure 2:**
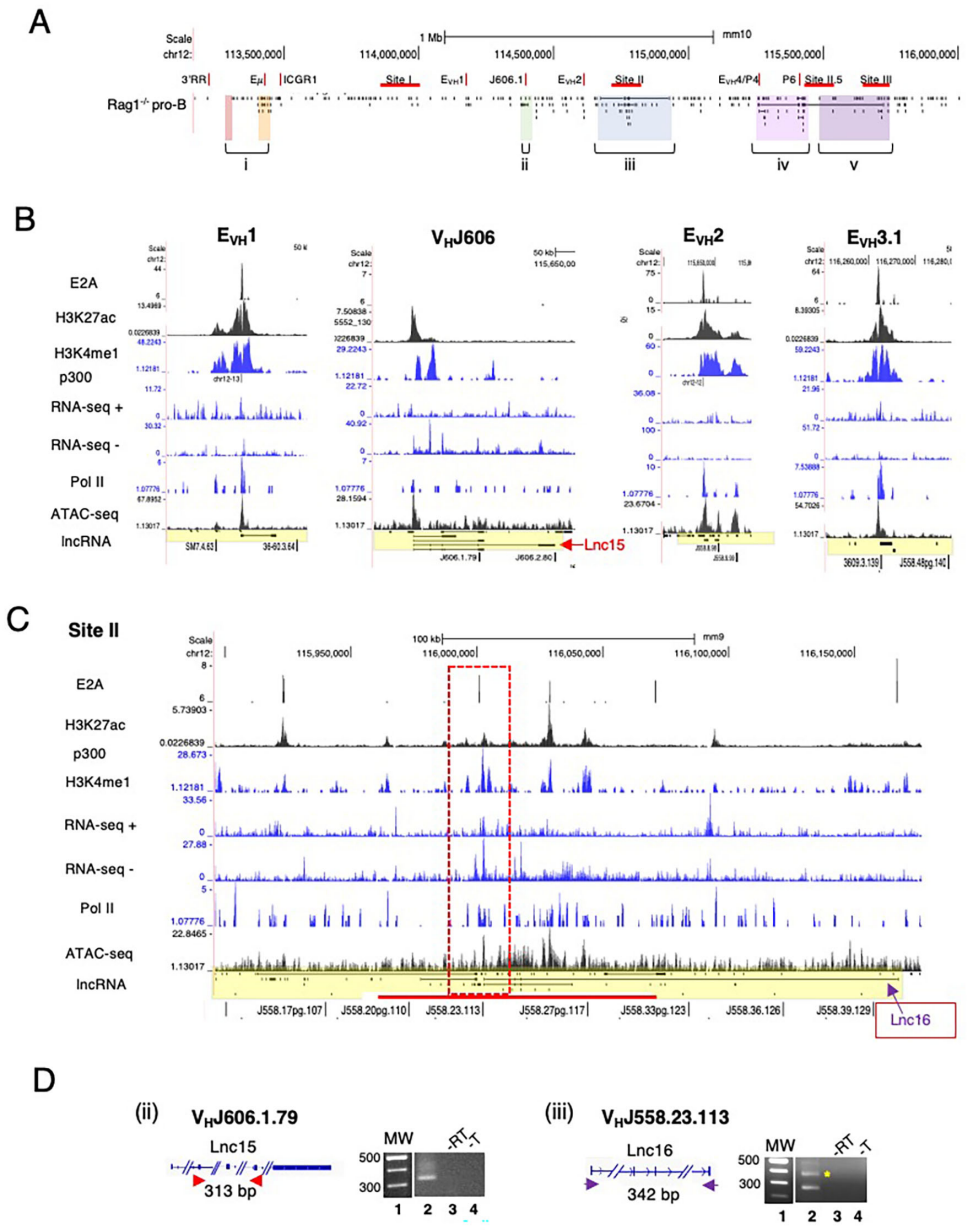
Spliced lncRNA are expressed from Igh enhancers and V_H_ promoters. **(A)** Schematic of the Igh locus shown with mono- and mutli-exonic lncRNAs arrayed below the map. **(B, C)** Multi-exonic lncRNAs in subTAD B (E_VH_1, V_H_J606.1.79 (V6-3), E_VH_2) and in Site II V_H_J558.23.113 (V1-23) of subTAD C are shown with lanes from ChIP-seq and RNA-seq, all from Rag deficient pro-B cells (UCSC browser mm9). **(D)** RT-PCR assays were performed. CDNA were derived from Rag2^-/-^ pro-B cells. MW 100bp ladder, no reverse transcriptase (-RT), no template (-T). Amplicons are diagrammatically indicated for each lncRNA. The predicted amplicon in panel iii is marked by the asterisk.

**Figure 3:**
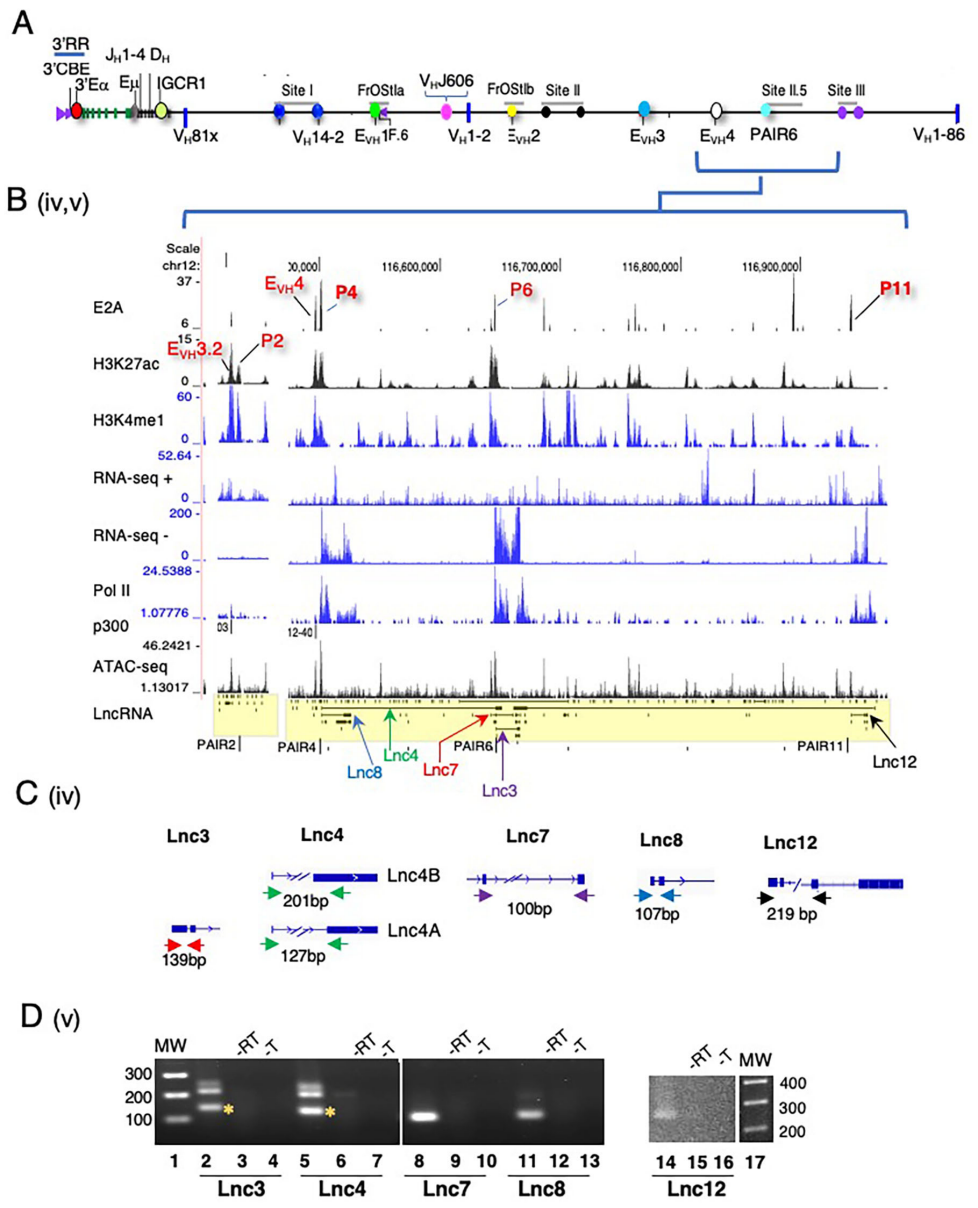
Multi-exonic lncRNA transcripts span the PAIR2-PAIR4-PAIR6-PAIR11 regions. **(A)** Schematic of the Igh locus. **(B)** Newly annotated multi-exonic lncRNAs centered on PAIR2, E_VH_4/PAIR4, PAIR6 at Site II.5 and PAIR11 at Site III are shown with lanes from ChIP-seq and RNA-seq from Rag deficient pro-B cells (UCSC browser mm9). **(C)** Amplicons are diagrammatically indicated for each lncRNA. **(D)** RT-PCR assays were performed. CDNA was synthesized using oligo dT and derived from Rag2^-/-^ pro-B cells. MW 100bp ladder, no reverse transcriptase (-RT), no template (-T). The predicted amplicon is marked by an asterisk.

To better appreciate the positional specificity of the multi-exonic lncRNA clusters we visualized these regions at high resolution using the UCSC Genome browser and provided an overall locus schematic for reference (**Figure 2A**). We observe that nearly all spliced lncRNAs are located at positions marked with H3K4me1 and H3K27ac which in combination is often indicative of active enhancer elements (70, 71). We performed ATAC-seq assays on Rag2^-/-^ pro-B cells to assess degrees of chromatin accessibility genome-wide. ATAC-seq signals indicate that multi-exonic lncRNAs TSSs are hyper-accessible in the Igh locus (**Figures 2B, C**, **3B**). We find that essentially all spliced and some mono-exonic lncRNAs are localized to Igh enhancers (Eγ, Eμ, E_VH_1, E_VH_2, E_VH_3.1, E_VH_3.2, E_VH_4) and occasionally at V_H_ promoters (**Figures 2B**, **3A, B**) (**Supplementary Figures 2A, B**). E_VH_3.2 and E_VH_4 exhibit an additional characteristic as they overlap with V_H_ promoters; V_H_8-6 (V_H_3609.5.147) adjacent to PAIR2, and V_H_8.7 (V_H_3609.6pg.151) near PAIR4, respectively (**Figure 1D**) (45). E_VH_3.1 is coincident with the TSS of a mono-exonic lncRNA that fully overlaps V_H_8-4 (V_H_3609.3.139). Spliced and nested lncRNAs also locate with other V_H_ promoters including V_H_6-3 (V_H_J606.1.79) and in S.II centered on V_H_1-23 (V_H_J558.23.113) (**Figures 2B, C**). High-throughput sequencing assays for enhancer activity have detected enhancer-like promoters that are located proximal to or overlapping with core promoters (72–74), anchor chromatin loops and function as *bona fide* enhancers (75). Our analyses highlight a convergence of lncRNAs with Igh enhancers and a subset of V_H_ promoters that harbor enhancer-like features.

#### 3.3.3 Spliced lncRNAs are actively transcribed in pro-B cells

We analyzed normalized expression (TPM) of mono-exonic and multi-exonic lncRNAs and found that many multi-exonic lncRNAs were expressed at levels significantly higher than for mono-exonic lncRNAs, (**Supplementary Figure 2C**). We verified expression of nine Igh associated multi-exonic lncRNAs (TPM >1.0) beginning with transcripts in the C_H_ domain including Cμ, μ0 (lnc1), Cγ2b, γ2b GLT (lnc9) in Rag2^-/-^ pro-B cells (**Supplementary Figure 2D**) (**Supplementary Table 1**). The novel multi-exonic lncRNAs, lnc15, from cluster ii (V_H_6-3, V_H_J606.1.79) and lnc16 in cluster iii (V_H_1-23, V_H_J558.23.113) were also confirmed (**Figure 2D**). The robust pattern of RNA-seq minus strand signals and Pol II binding at PAIR4, PAIR6 and PAIR11 closely corresponds with the exon layout for lnc8, lnc7 and lnc12 suggesting active transcription (**Figures 3A, B**).

LncRNAs are often expressed as RNA isoforms which are produced via alternative transcription start and poly(A) sites and by alternative splicing (76, 77) resulting in varied transcripts (78). Many of the Igh associated multi-exonic lncRNAs are nested and appear to originate from alternative transcription starts and/or from alternative splicing (**Figures 2A, B**, **3B**). Using polyA+ RNA for cDNA synthesis we demonstrate here that lnc3, lnc4, lnc7, lnc8 and lnc12 are polyadenylated in RT-PCR assays of pro-B cells (**Figures 3C, D**). LncRNAs lnc4 and lnc8, are splice variants located at PAIR4 and initiate from a single TSS (**Figures 3C, D**). The additional PCR product found for lnc4 originates from an overlapping mono-exonic transcript. Polyadenylation of lnc8 originating from PAIR4 was previously shown (66). PAIR6 associated lnc3 and lnc7 are isoforms with different TSSs and lnc12 is located at PAIR11 (**Figures 3C, D**). The primers for Lnc3 amplification overlap with a mono-exonic lncRNA and PCR generates two products, one 127bp (indicated by the asterisk) and a second larger product (**Figure 3D**). Lnc7 initiates downstream of V_H_8-8-1 (V_H_3609.8pg.160) and completely overlaps this gene whereas lnc3 initiates at PAIR6. Our studies have confirmed the expression of several newly Igh associated nested lncRNA transcripts.

### 3.4 Site II anchors long range interactions with other Igh structural elements

**Figure 4:**
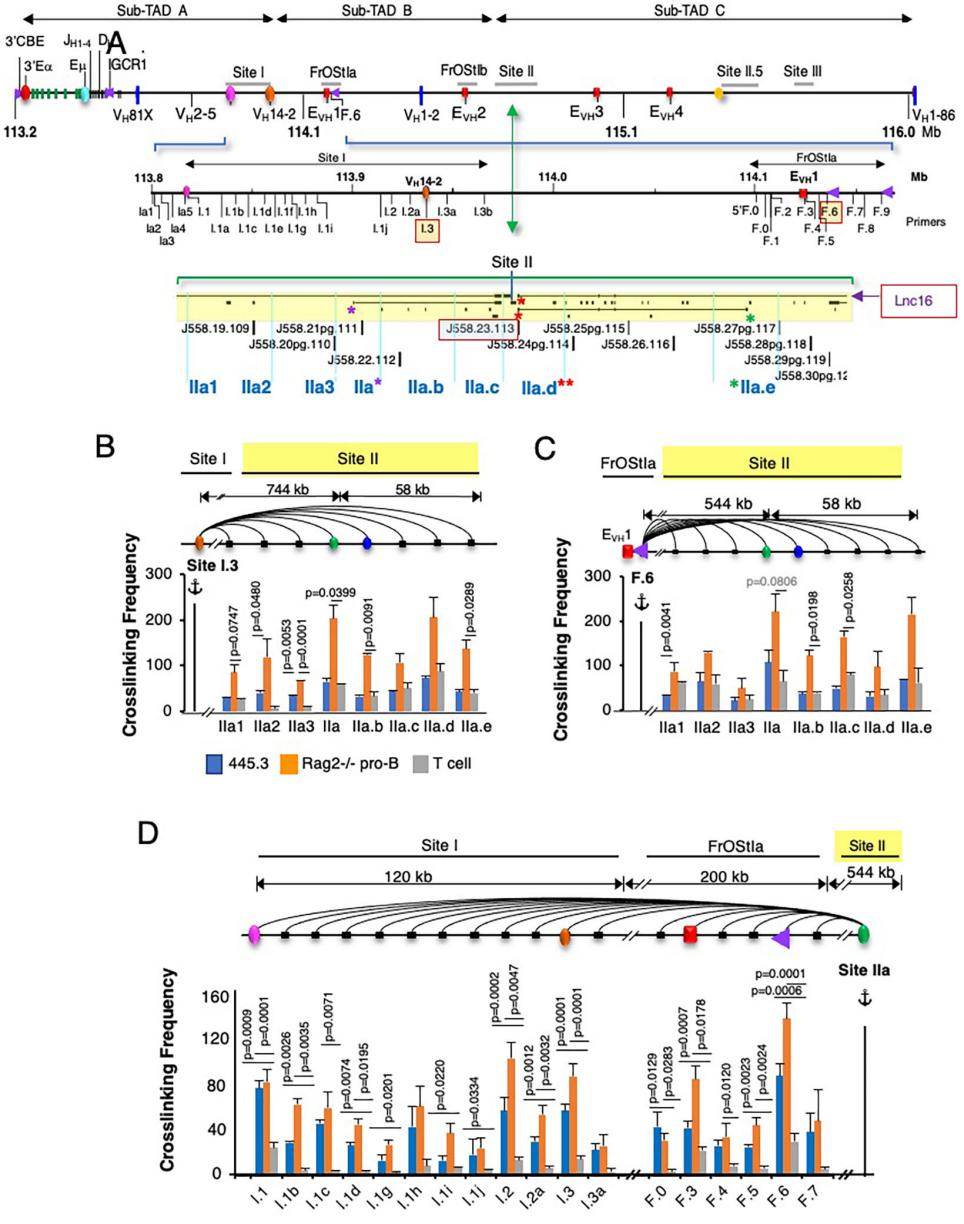
Identification of Site II loop anchors locations. **(A)** Schematic of the Igh locus with genomic coordinates (chr12, mm10) the directionality of which follows chromosome showing DNA elements (CBEs, purple arrows, E_VH_1, E_VH_2, E_VH_3, E_VH_4 (red ovals). 3C primer sites are indicated for Site I and FrOStIa (middle panel). Site II is expanded, 3C Hind III fragments are delineated (vertical teal lines) and primer sites named (bottom panel). 3C fragments IIa, IIa.d and IIa.e and the lncRNAs that reside within them are marked by asterisks. **(B-D)** 3C assays. Arcs (3C assays), primers are identified below the graphs, 3C probes (anchor symbol). Average crosslinking frequencies are from independent chromatin samples as indicated. Statistical comparisons are to T cells. P values from two-tail Student’s t test and SEMs are shown. **(B, C)** 3C assays analyzing Site II anchored at Site I.3 **(B)** and FrOStIa F.6 CBE **(C)**. Chromatin samples for Abl-t 445.3.11 line, n=3; Rag2^-/-^ pro-B cells, n=3; T cells: n=3. **(D)** 3C assays analyzing Site I and FrOStIa anchored at Site II **(D)**. Chromatin samples for Abl-t 445.3.11line, n=3; Rag2^-/-^ pro-B cells, n=3; T cells: n=3.

To examine the relationship of lncRNA expression with chromatin loop anchors in greater detail we analyzed S.II for Igh looping interactions in 3C assays. The spatial organization of Igh subTADs A and B is sculpted in part by interactions between the S.I V_H_14–2 promoter with Eμ, and with FrOStIa E_VH_1 and F.6 (**Supplementary Figure 1**) (45). V_H_14–2 is the most highly transcribed V_H_ gene in the locus (4, 5) and is located on the S.I.3 3C fragment (45). E_VH_1 is a modulator of regional V_H_ transcription and gene usage during V->DJ recombination and is a structural anchor of subTAD B (45). F.6 is bound by CTCF, is located ~15 kb upstream of E_VH_1 and interacts with V_H_14-2 (**Figure 4A**) (45). Using anchor probes S.I.3 and F.6 located in subTADs A and B, respectively, we characterized points of chromatin contact at S.II in chromatin from Rag2^-/-^ pro-B cells, the Abelson transformed (Abl-t) pro-B cell line, 445.3, and ConA activated splenic T cells (**Figure 4A**). We anticipated that long range contacts between S.II and interaction partners in subTADs A and B, will occur in Rag2^-/-^ pro-B cells which engage in robust Igh locus contraction, less in Abl-t 445.3 pro-B-cells that are partially deficient in locus contraction and will be absent in T cells (21, 58). To facilitate detection of extremely long-range contacts 3C chimeric fragments underwent a pre-amplification PCR step which was then used to program 3C assays.

Several points of statistically significant chromatin contact were detected for the S.I.3 and F.6 anchor probes with S.II in Rag2^-/-^ pro-B cells but not in the 445.3 cell line or in splenic T cells indicating that these interactions depend on locus contraction (**Figures 4B, C**). S.I.3 and F.6 anchors prominently and reproducibly interact with fragments S.IIa and S.IIa.b and more sporadically with other fragments throughout this region (**Figures 4B, C**). S.II fragments IIa, IIa.d and IIa.e contain the TSSs and/or termination points of four nested lncRNAs (marked by red, green and purple asterisks), including lnc16, that is centered on V1-23 (V_H_J558.23.113) (**Figures 2C**, **4A** lower panel). Next, we assessed the S.IIa anchor contacts with S.I and FrOStIa and found a series of statistically significant interactions across these regions that were robustly present in in Rag2^-/-^ pro-B cells, absent in splenic T cells and present at low levels in the 445.3 pro-B cell line highlighting the importance of locus contraction for formation of very long-range contacts (**Figure 4D**). The S.IIa probe contact patterns were particularly robust for S.I.2 and S.I.3, and FrOStIa F.3 (E_VH_1) and F.6, in pro-B cells recapitulating earlier studies identifying these sites as major loop tethers (**Figure 4D**) (45). We conclude that S.II anchors long range interactions at positions coincident with multi-exonic lncRNA expression. We note that lncRNAs are also expressed at E_VH_1 and within the SI.3 3C fragment. Thus, lncRNAs are positionally correlated with chromatin loop anchors that support locus topology. A limitation of our study is that analysis of mutations that would define the precise lncRNA gene elements required for chromatin loop function has not yet been carried out. Nevertheless, the correlation of lncRNA position with chromatin anchors is tight.

## 4 Discussion

LncRNAs are diverse transcriptional products emerging from thousands of loci with numerous functions and the potential to regulate gene expression at varying distances from their targets (reviewed in (30–32, 79)). Antisense intergenic and genic transcripts in the V_H_ domain of the Igh locus were previously detected and their expression was concomitant with V->DJ recombination in pro-B cells (80). More recently the involvement of lncRNAs was shown in early B cell development, V(D)J recombination and class switch recombination (CSR) (37, 38). Our interrogation of unannotated sense and antisense transcripts (>300nt) genome-wide revealed a remarkable enrichment of previously unannotated lncRNAs in the Igh locus of pro-B cells and high cell specific expression in AgR loci that is correlated with the onset of V(D)J recombination. We find that Igh associated lncRNAs are far more pervasive than originally contemplated. Multi-exonic lncRNAs are focused to Igh enhancers, a subset of V_H_ promoters with enhancer-like features and which appear to function as chromatin loop anchors that define locus topology.

Two forms of transcription products originating from enhancers have been detected, enhancer RNAs (eRNAs) and lncRNAs. Active enhancers display characteristic chromatin marks including H3K37ac, H3K4me1, bind CBP/p300, coincide with DNase hypersensitive sites (81) and exhibit extensive transcription leading to the production of eRNAs which are typically short, unstable, non-polyadenylated and unspliced (82–86). RNAPII binding and bidirectional eRNA expression are now considered hallmarks of active enhancers (84, 85). Unlike eRNA, lncRNAs are generally stable, spliced and polyadenylated (85, 87) as are many Igh associated multi-exonic transcripts. Enhancers containing conserved, directional splicing signals that promote lncRNA production often exhibit elevated activity and implicate lncRNA processing as a factor determining enhancer function (88). Genomewide annotation studies have shown that 30-60% of lncRNAs are transcribed from positions with characteristic enhancer features (85, 89). Notably, Igh associated spliced lncRNAs are overlapping with or immediately adjacent to seven Igh enhancers (Eγ, Eμ, E_VH_1, E_VH_2, E_VH_3.1, E_VH_3.2, E_VH_4) which also anchor loop domains. Comparative studies indicate that lncRNA associated enhancers comprise a subgroup with stronger enhancer activity than those unaffiliated with lncRNAs (88) implying that Igh enhancers operate at an elevated activity level. Interestingly, recent studies reported that splicing of coding and noncoding RNA transcripts could increase expression of nearby genes (72, 90). Extrapolation of these findings to the Igh locus suggests that expression of lncRNAs could influence nearby V_H_ gene expression and perhaps their usage in V(D)J recombination.

Multi-exonic lncRNA genes are also associated with a subset of V_H_ genes including V_H_6–3 in subTAD B and at S.II centered on V_H_1–23 which exhibit elevated chromatin accessibility and H3K4me1 and H3K27ac marks, an epigenetic signature of enhancers. The distal enhancers E_VH_3.1, E_VH_3.2 and E_VH_4 are co-located with V_H_ promoters and all overlap lncRNAs. Accordingly, enhancer activity has been detected at a subgroup of promoters with enhancer-like characteristics that are positioned at or near core promoters (72–74), and function as *bona fide* enhancers and chromatin loop anchors (75). Our chromosome conformation capture studies presented here and in earlier work (45) have shown that the Igh enhancers and enhancer-like promoters are configured in a long-range multiway hub that contributes to locus conformation and is topologically linked with Eμ (45). Deletion of E_VH_1, an enhancer hub constituent, led to altered locus topology and regional loss of V_H_ gene transcription and reduced V_H_ usage in V->DJ recombination (45). Our findings indicate that a subset of Igh associated lncRNAs are embedded in an enhancer cluster with likely importance to locus function. Our findings raise the question of how lncRNA expression might influence genome architecture. Accordingly, studies indicate that lncRNAs can influence chromatin function and regulate membraneless nuclear bodies, for example (91).

Genome folding is catalyzed through cohesin mediated loop extrusion, a major contributor toward shaping the spatial organization of the genome (16, 17, 19, 28, 92–95). Cohesin maintains TADs by progressively extruding DNA loops until it becomes encumbered by an obstacle which halts movement and anchors DNA loops in mammalian cells (16, 17). The loop extrusion model explains how Es can processively track arrays of Prs that are separated by long genomic intervals (16, 17, 96). Loop extrusion can be blocked by chromatin binding proteins leading to the generation of cohesin dependent loops.

CTCF is the most prominent loop extrusion barrier (28, 94, 95) and RNAPII and R-loops play similar roles at active promoters (97, 98). For example, RNAPII depletion leads to the establishment of longer chromatin loops (99) and reorganization of contacts between cohesin mediated CTCF anchored loops (100) indicating a dynamic relationship of RNAPII with loop extrusion. The Igh V_H_ domain contains 144 CTCF binding elements (CBEs) that are occupied by CTCF in pro-B cells (8, 101) implying a substantial role for CTCF in specifying locus structure. Indeed, deregulation of CBE impediments in primary pro-B cells promotes V(D)J recombination mediated by loop extrusion (23). Likewise, RNAP II can directly tether chromatin loops (100, 102) and contribute to cohesin pausing (103, 104). Exuberant expression of lncRNAs in the Igh locus of pro-B cells and the pile-up of both mono- and multi-exonic lncRNAs at loop anchors implies that clustered RNAPII binding may cumulatively impede cohesin mediated loop extrusion and create logjams leading to emergence of major loop anchors.

V_H_ gene families are clustered in the locus (3) and display distinct epigenetic signatures defined by histone modifications, RNAPII and TF binding (4, 5) and the position of bound CTCF (7, 8). Clustering of Igh associated lncRNA genes will produce foci of RNAPII binding which could obstruct loop extrusion as has been found in other loci (98). We propose that the dynamic interplay of loop extrusion with its barriers, CTCF, and RNAPII including those loaded at lncRNA promoters may modulate the contact probability of V_H_ genes with the recombination center and influence V_H_ gene usage during recombination.

## Data Availability

The datasets presented in this study can be found in online repositories. The names of the repository/repositories and accession number(s) can be found in the article/**Supplementary Material**.
