# Supplementary material for: Exuberant long noncoding RNA expression may sculpt Igh locus topology
Source: Front Immunol. 2025 Nov 24;16:1678105. doi: 10.3389/fimmu.2025.1678105 (PMC12682764; doi:10.3389/fimmu.2025.1678105)
Supplement: Supplementary file 1 [file Presentation1.pptx]

## Slide 1
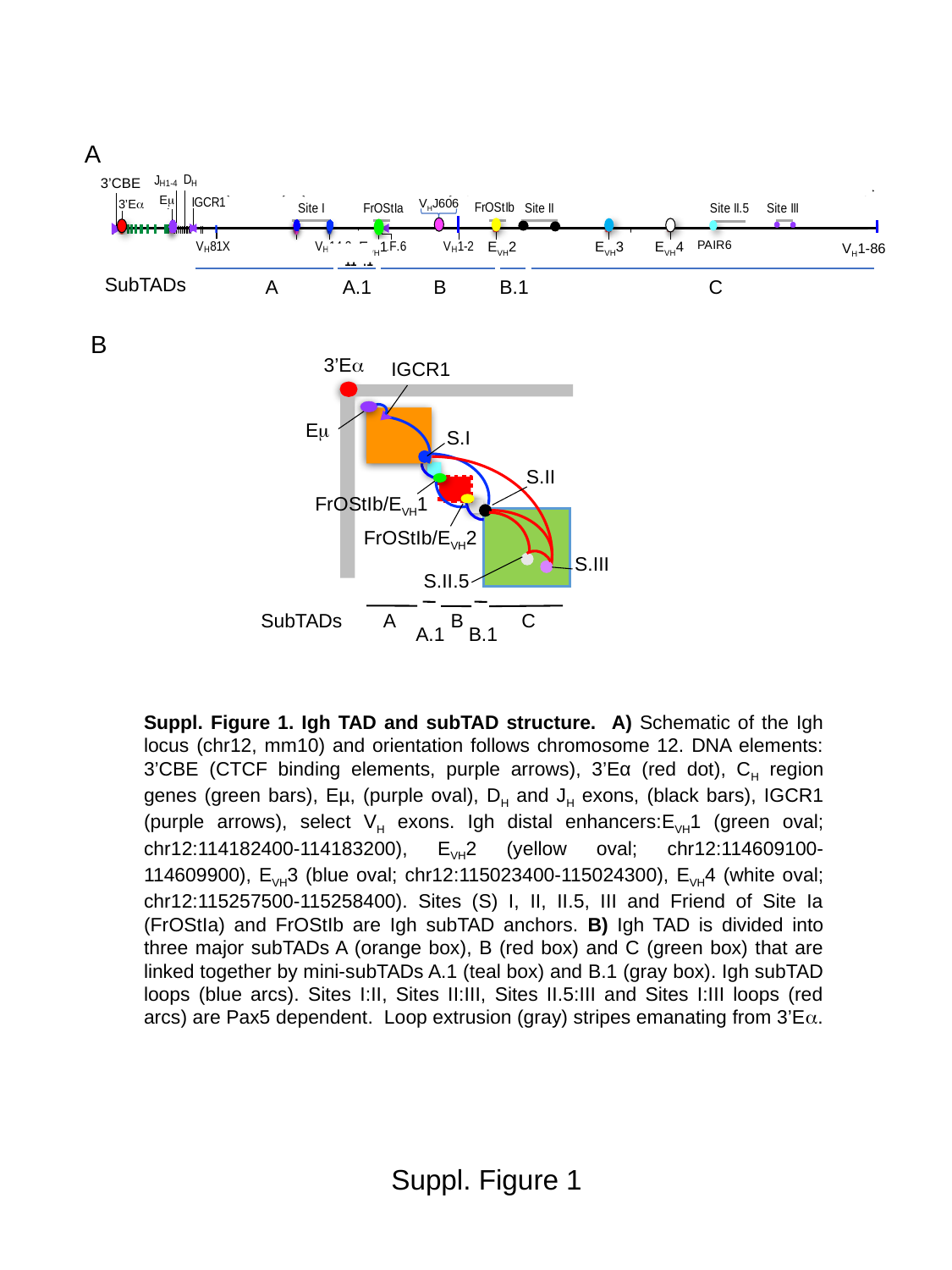

A
SubTAD A
SubTAD C
SubTAD B
3’CBE
Em
VHJ606
3’Ea
.
.
PAIR6
EVH1
EVH2
EVH3
EVH4
VH1-86
SubTADs
A
A.1
B
B.1
C
3’CBE
B
3’Ea
IGCR1
Em
S.I
S.II
FrOStIb/EVH1
FrOStIb/EVH2
S.III
SubTADs
A
B
C
A.1
B.1
S.II.5
Suppl. Figure 1. Igh TAD and subTAD structure. A) Schematic of the Igh locus (chr12, mm10) and orientation follows chromosome 12. DNA elements: 3’CBE (CTCF binding elements, purple arrows), 3’Eα (red dot), CH region genes (green bars), Eµ, (purple oval), DH and JH exons, (black bars), IGCR1 (purple arrows), select VH exons. Igh distal enhancers:EVH1 (green oval; chr12:114182400-114183200), EVH2 (yellow oval; chr12:114609100-114609900), EVH3 (blue oval; chr12:115023400-115024300), EVH4 (white oval; chr12:115257500-115258400). Sites (S) I, II, II.5, III and Friend of Site Ia (FrOStIa) and FrOStIb are Igh subTAD anchors. B) Igh TAD is divided into three major subTADs A (orange box), B (red box) and C (green box) that are linked together by mini-subTADs A.1 (teal box) and B.1 (gray box). Igh subTAD loops (blue arcs). Sites I:II, Sites II:III, Sites II.5:III and Sites I:III loops (red arcs) are Pax5 dependent. Loop extrusion (gray) stripes emanating from 3’Ea.
Suppl. Figure 1

## Slide 2
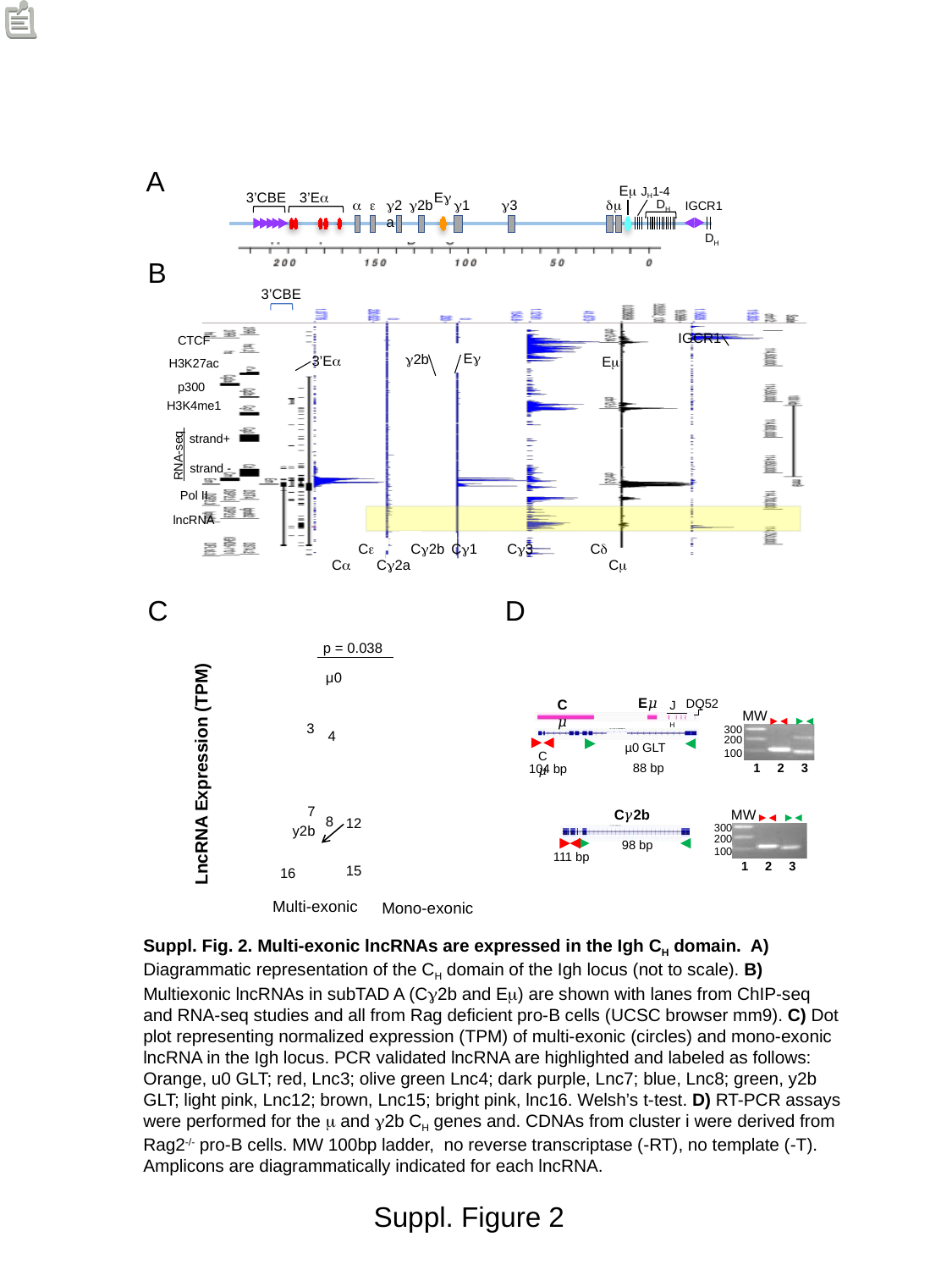

A
Em
JH1-4
3’CBE
3’Ea
Eg
a
e
g2a
g2b
g1
g3
d
m
DH
IGCR1
DH
3’CBE
IGCR1
CTCF
Eg
g2b
3’Ea
Em
H3K27ac
p300
H3K4me1
 strand+
RNA-seq
strand -
Pol II
lncRNA
Ce
Cg2b
Cg1
Cg3
Cd
Ca
Cg2a
Cm
B
C
D
p = 0.038
μ0
3
4
LncRNA Expression (TPM)
7
8
12
y2b
15
16
Multi-exonic
Mono-exonic
E𝜇
C𝜇
DQ52
JH
µ0 GLT
C𝜇
88 bp
104 bp
300
200
100
1 2 3
MW
MW
C𝛾2b
98 bp
111 bp
300
200
100
1 2 3
Suppl. Fig. 2. Multi-exonic lncRNAs are expressed in the Igh CH domain. A) Diagrammatic representation of the CH domain of the Igh locus (not to scale). B) Multiexonic lncRNAs in subTAD A (Cg2b and Em) are shown with lanes from ChIP-seq and RNA-seq studies and all from Rag deficient pro-B cells (UCSC browser mm9). C) Dot plot representing normalized expression (TPM) of multi-exonic (circles) and mono-exonic lncRNA in the Igh locus. PCR validated lncRNA are highlighted and labeled as follows: Orange, u0 GLT; red, Lnc3; olive green Lnc4; dark purple, Lnc7; blue, Lnc8; green, y2b GLT; light pink, Lnc12; brown, Lnc15; bright pink, lnc16. Welsh’s t-test. D) RT-PCR assays were performed for the m and g2b CH genes and. CDNAs from cluster i were derived from Rag2-/- pro-B cells. MW 100bp ladder, no reverse transcriptase (-RT), no template (-T). Amplicons are diagrammatically indicated for each lncRNA.
Suppl. Figure 2
