# Supplementary material for: Exuberant long noncoding RNA expression may sculpt Igh locus topology
Source: Front Immunol. 2025 Nov 24;16:1678105. doi: 10.3389/fimmu.2025.1678105 (PMC12682764; doi:10.3389/fimmu.2025.1678105)
Supplement: Supplementary file 2 [file Table1.docx]

**Exuberant long noncoding RNA expression may sculpt Igh locus topology**

Ellen B. Drake^1^, Sarah Naiyer^1,4^, Xinyan Qu^1,5^, Khalid Bhat^1,6^, Hammad Farooq^2^, Mark Maienschein-Cline ^3^ Jie Liang^2^ and Amy L. Kenter^1,*^

**Supplementary Tables 1-3**

**Supplementary Table 1. Primers for LncRNA RT-PCR assays**

| **Amplicon** | **Primer Name** | **Sequence** |
| --- | --- | --- |
| μ0 GLT | μ0 exon1F | GAACAGAGGCAGAACAGAGAC |
|  | μ0 exon2R | GAAGACATTTGGGAAGGACTGA |
| Cμ region | μ0 exon6F | TCCACCTTCATCGTCCTCTT |
|  | μ0 exon7R | CCTTCCATGCTGAGAGTCATTT |
| Lnc3 | Lnc3.E1-2F | CCTGTTCCCTGTGTCTTCAA |
|  | Lnc3.E1-2R | CATGACTATTGGGAGGTGAGAG |
| Lnc4 | Lnc4.E1-2F | GTCTTCAAAGCTCCATGTTAGTG |
|  | Lnc3.E1-2R | CATGACTATTGGGAGGTGAGAG |
| Lnc7 | Lnc7.E2-3F | AGGCTCTCACCTCCCAATA |
|  | Lnc7.E2-3R | GACACTCACCTGGAAACTGAA |
| Lnc8 | Lnc7.E2-3F* | AGGCTCTCACCTCCCAATA |
|  | Lnc8.E2-3R | TCATTGAACATCACGGAGAGAA |
| γ2b GLT | γ2b exon1F | CACACCTACAGACAACCAGAC |
|  | γ2b exon2R | ATCCCAGAGTCACAGAGGAA |
|  | γ2b exon4F | AACAAAGACCTCCCATCACC |
|  | γ2b exon5R | TTTCCTGGACAACTGCTCTG |
| Lnc12 | 115703F | GAGCTCTGCTTCAGAATGAACTA |
|  | 115714R | CCTTCTACCTGCCTTTGATGAG |
| Lnc15 | 114382F | CACTTCCCAACACTTCCTCTAA |
|  | 144403R | GCACATGGACACAGTGGAA |
| Lnc16 | 114764F | GGGATTGGACACCTATGAAGAA |
|  | 114929R | CCTGATATGGCTATCTCCTGAAA |
| *Lnc7 F primer also anneals to lnc8 exon2 due to sequence homology. Lnc7 and lnc8 do not overlap. | | |

**Supplementary Table 2. In situ Hi-C Library Statistics.**

|  | **Rag2^-/-^1** | **Rag2^-/-^2** | **Rag2^-/-^_merged_replicates** |
| --- | --- | --- | --- |
| **Sequenced Read Pairs** | 1,043,441,952 | 1,478,311,863 | 2,521,753,815 |
| **Normal Paired** | 331,289,893 (31.75%) | 453,536,262 (30.68%) | 784,826,155 (31.12%) |
| **Chimeric Paired** | 592,636,236 (56.80%) | 852,524,198 (57.67%) | 1,445,160,434 (57.31%) |
| **Chimeric Ambiguous** | 115,369,368 (11.06%) | 165,080,822 (11.17%) | 280,450,190 (11.12%) |
| **Unmapped** | 4,146,455 (0.40%) | 7,170,581 (0.49%) | 11,317,036 (0.45%) |
| **Alignable (Normal+Chimeric Paired)** | 923,926,129 (88.55%) | 1,306,060,460 (88.35%) | 2,229,986,589 (88.43%) |
| **Unique Reads** | 726,007,667 (69.58%) | 973,960,901 (65.88%) | 1,685,950,359 (66.86%) |
| **PCR Duplicates** | 190,125,933 (18.22%) | 320,232,444 (21.66%) | 524,529,621 (20.80%) |
| **Optical Duplicates** | 7,792,529 (0.75%) | 11,867,115 (0.80%) | 19,506,609 (0.77%) |
| **Library Complexity Estimate** | 1,889,922,420 | 2,163,177,142 | 3,887,353,521 |
| **Below MAPQ Threshold** | 133,564,985 (12.80% / 18.40%) | 183,580,146 (12.42% / 18.85%) | 315,337,363 (12.50% / 18.70%) |
| **Hi-C Contacts** | 592,442,682 (56.78% / 81.60%) | 790,380,755 (53.47% / 81.15%) | 1,370,612,996 (54.35% / 81.30%) |
| **Inter-chromosomal** | 63,963,999 (6.13% / 8.81%) | 81,980,226 (5.55% / 8.42%) | 145,939,275 (5.79% / 8.66%) |
| **Intra-chromosomal** | 528,478,683 (50.65% / 72.79%) | 708,400,529 (47.92% / 72.73%) | 1,224,673,721 (48.56% / 72.64%) |
| **Short Range (<20Kb)** | 177,046,539 (16.97% / 24.39%) | 240,762,563 (16.29% / 24.72%) | 405,638,486 (16.09% / 24.06%) |
| **Long Range (>20Kb)** | 351,408,243 (33.68% / 48.40%) | 467,610,642 (31.63% / 48.01%) | 818,984,731 (32.48% / 48.58%) |

**Supplementary Table 3. Primers and probes for 3C assays**

| **Primer** | **TagMan**  **Probe** | **Sequence** | **Reference** |
| --- | --- | --- | --- |
| Ia1 |  | GGAAGGGAATGGGCATTAATGCATTAAG | (6) |
| Ia2 |  | CAGCTGTAGTGATGGTAATAAAGTG | (6) |
| Ia3 |  | CGAGTAAATGGGGACAGGGGAAAT | (6) |
| Ia4 |  | CTTAGCCTTTCGGTATTCCTCC | (6) |
| Ia5 |  | GGTATCCAGAACTTGATGTGGTGA | (6) |
| I.1 |  | GTCGTCATCTACACACAGGAGC | (6) |
| I.1a |  | GAAAACAAACTCAAAGACAAACTACATGATA | (6) |
| I.1b |  | CCAGATACATAGAATAGGAGAGAAGAC | (6) |
| I.1c |  | CGGAGAAGATCAAATTGAAAATCCTCTC | (6) |
| I.1d |  | GGACCCATCCAACCATCTTTGAAATG | (6) |
| I.1e |  | GACTGTTGTTGACAGATTTGGCATTC | (6) |
| I.1f |  | CTGGACTGTAAAGCCAGTTCCAC | (6) |
| I.1g |  | CTGGAGTCTGGCACCATCATCAT | (6) |
| I.1h |  | GATAAAACTGAAGGTCTGCCCACTC | (6) |
| I.1i |  | CTCTCTGAGCTCAGGAATTCCAG | (6) |
| I.1j |  | CAAGAACAAATTTGAGTCAGATCTTCTG | (6) |
| I.2 |  | CGTTATGCTTTTAATTTGCTCACTTATAAG | (6) |
| I.2a |  | CAATACCCATATGGTATTTTCAACAATTTTTG | (6) |
| I.3 |  | TCCTACTTCTGTCTGAGGAAATTTCG | (6) |
| I.3a |  | CTCAGGAATTCGAGCAGAAGAT | (6) |
| I.3b |  | ACAAACTCTGAAGAAGGTCCGAAGGT | (6) |
|  | **P_Site I.3** | TGATATTGTATCTAAGTGGTCTGCACATGTCTCAG | (6) |
| 5’F.0 |  | ATTCTTCTCAATCCTGGGACTAGT | (6) |
| F.0 |  | TGTCTGCATGGTGTTTCTTTCATAC | (6) |
| F.1 |  | TAGTGCAGCTTCCACTTAAAGAAC | (6) |
| F.2 |  | GACACCATGGAGATGGATCATC | (6) |
| F.3 |  | CCTCCTGCAGCAGGGTTATTC | (6) |
| F.4 |  | CCCACAAATGCCAGACTAAAGAAC | (6) |
| F.5 |  | CACTTTGAAGCTGAAGACATTGAC | (6) |
| F.6 |  | CTGAAAGCAGGCTATTCACCATG | (6) |
| F.7 |  | GCAAAGGACCTCTAAAGGATTGC | (6) |
| F.8 |  | GATTCCATAAACAGAACCCTAATGG | (6) |
| F.9 |  | GCCTGTTTGTTGAGAAGTGCACA | (6) |
|  | **P_F.6** | AGAGCAACAAGGAAAAGCCATCTAAGCTCCAA | (6) |
| Fb.1 |  | TTACCCACTCTATCCAGTAGCC | (6) |
| Fb.2 |  | CGTTCAGAGCAACACTGCCCTA | (6) |
| Fb.3 |  | ACTTGGAGACATCTAAGAAAAGTAAGATTAG | (6) |
| Fb.4 |  | CTTAGAGGACTACTTTCATAATTGAATTCC | (6) |
| Fb.5 |  | ATCCCTACTCTTGAATCTCAATGAGG | (6) |
| Fb.6 |  | ATTTGCAGAAAGTTGAGTAGAAAGAACAG | (6) |
| Fb.7 |  | CGTTTGTGCTATATGATAGAGATTTGCCA | (6) |
| Fb.8 |  | CTGCTAGAGTAGCCCTTGTCCATAA | (6) |
| Fb.9 |  | GTGAACATTACCACATGAGGGACTTC | (6) |
| IIa1 |  | CATCAAGTATCAATAAAAGAGACCTCATAAAG | (6) |
| IIa2 |  | GAAACGCCAATCTGACTTCCAGTG | (6) |
| IIa3 |  | AGAGAAACACTCCTCCACTTCCG | (6) |
| IIa |  | GATGTGTAACTGTCTCCGTAATAATATATAG | (6) |
| IIa.b |  | CATCCCAGACAGAGCTACAGAG | (6) |
| IIa.c |  | TGGGAGAAGTTGCACTTACATCTTG | (6) |
| IIa.d |  | ACTCTTATATTCTAAAAACCTGGAACTTG | (6) |
| IIa.e |  | TCAAAGCAACCCTAAGAATCTCCTTC | (6) |
|  | **P_Site IIa** | TCTTCCCTCCCAAGCATGTACTCA | (6) |
| GD F |  | AGGCTTCTGACCTGCATCTTGA | (7) |
| GD R |  | TTCCAGAGCATTGTCAGCAAA | (7) |
|  | **GD** | ACCTTGCTACTCTTCCCTGGTGTTTGTTGG | (7) |
| Mb1 F |  | CCACGCACTAGAGAGAGACTCAA | (6) |
| Mb1 R |  | CCGCCTCACTTCCTGTTCAGCCG | (6) |
